# Sarcoidosis and Autoimmune Inflammatory Syndrome Induced by Adjuvants

**DOI:** 10.3390/life13041047

**Published:** 2023-04-19

**Authors:** Anna Starshinova, Yulia Zinchenko, Anna Malkova, Dmitriy Kudlay, Igor Kudryavtsev, Piotr Yablonskiy

**Affiliations:** 1 Almazov National Medical Research Centre, 197341 Saint-Petersburg, Russia; igorek1981@yandex.ru; 2 Saint-Petersburg Research Institute of Phthisiopulmonology, 194064 Saint-Petersburg, Russiapiotr_yablonskii@mail.ru (P.Y.); 3 Laboratory of the Mosaic of Autoimmunity, Saint-Petersburg State University, 199034 Saint-Petersburg, Russia; anya.malkova.95@mail.ru; 4 Medical Department, Sechenov First Moscow State Medical University, 119435 Moscow, Russia; 5 Institute of Immunology, 115478 Moscow, Russia; 6 Department of Immunology, Institution of Experimental Medicine, 197022 Saint-Petersburg, Russia

**Keywords:** sarcoidosis, autoimmunity, ASIA, adjuvants, Heerfordt’s syndrome, Löfgren’s syndrome, bacteria

## Abstract

Currently, sarcoidosis remains one of the diseases with unknown etiology, which significantly complicates its diagnosis and treatment. Various causes of sarcoidosis have been studied for many years. Both organic and inorganic trigger factors, provoking the development of granulomatous inflammation are considered. However, the most promising and evidence-based hypothesis is the development of sarcoidosis as an autoimmune disease, provoked by various adjuvants in genetic predisposed individuals. This concept fits into the structure of the autoimmune/inflammatory syndrome, induced by adjuvants (ASIA) that was proposed in 2011 by Professor Shoenfeld Y. In this paper, the authors reveal the presence of major and minor ASIA criteria for sarcoidosis, propose a new concept of the course of sarcoidosis within the framework of ASIA, and point out the difficulties in creating a model of the disease and the selection of therapy. It is obvious that the data obtained not only bring us closer to understanding the nature of sarcoidosis, but also potentiate new studies confirming this hypothesis by obtaining a model of the disease.

## 1. Introduction

According to the current concept, sarcoidosis occurs as a result of exposure to various exogenous or endogenous antigen factors in subjects with a genetic predisposition to autoimmune disorders, and is associated with the development of non-caseating granulomas in different organs [1].

Sarcoidosis is one of the granulomatous diseases with an acute or chronic course. Inflammation most often involves the lungs and mediastinal lymph nodes in up to 90% of cases, as well as other organs and tissues, with granuloma formation without caseous necrosis. Currently, there are two variants of the sarcoidosis acute course: Lofgren’s and the Heerfordt–Waldenström syndrome [2].

The search for etiologic factors of sarcoidosis has revealed various infectious agents (from bacteria to viruses and fungi), as well as non-organic factors (e.g., silicone, silicates etc.) that could be associated with its development [3].The problem of finding an etiological factor in studies on sarcoidosis has led to the identification of various infectious agents from bacteria to viral agents and fungi and inorganic factors (silicone, silicates, etc.) [3,4]. The lack of an etiological factor and understanding of the pathogenesis of the disease leads to the absence of a unified approach to therapy, as well as to the possibility of conducting preclinical studies of the effectiveness of treatment on sarcoidosis models [5].

The main key of the pathogenesis of sarcoidosis is the formation of granulomas in the lungs, mediastinal lymph nodes, skin, and other organs [1].

In sarcoidosis, patients with a genetic predisposition to this disease, in which antigen-presenting cells (macrophages, dendritic cells, epithelial cells), come into contact with an unknown foreign antigen results in the dysregulated immune response that manifests in granulomatous inflammation [6].

The main characteristic of sarcoidosis is the formation of noncaseating epithelioid granulomas in various organs, represented by lymphocytes, epithelioid, and giant cells (asteroid and conchoidal bodies). Unlike infectious-mediated granulomas in sarcoidosis, necrotic masses are not formed and ACE (*Angiotensin-converting enzyme 2*) hyperproduction occurs [7]. In recent years, the number of proponents of the autoimmune nature of the theory of this disease increases. This theory has been confirmed by numerous studies [7,8]. The central part of a granuloma is composed of macrophages, modified macrophages, epithelioid cells, and giant cells, with CD4+ T-lymphocytes between them [8]. The peripheral part of a granuloma is predominantly occupied by CD8+ T lymphocytes, fibroblasts, macrophages, and fibrocytes. B lymphocytes are usually very rare in a granuloma [9]. Macrophages, dendritic cells, and epithelial cells are the first cells to meet the antigens due to the presence of Toll-like receptors. The long-term exposure of Toll-like receptors to foreign antigens results in the activation and epithelioid differentiation of macrophages, which start producing proinflammatory cytokines (TNF-a, IL-1). Having bound to the antigen, dendritic cells migrate to the lymph nodes, where they present the antigen to T-lymphocytes [10]. The accumulation of epithelioid macrophages and T- and B-lymphocytes in the locus of inflammation leads to the formation of epithelioid granulomas without the foci of caseous necrosis. Later, there is cellular damage resulting from the effectors of both the humoral (antibodies) and cellular immune response (cytokines of T-lymphocytes). In the chronic form, fibrosis occurs due to Tx17 and TNF-ά that is synthesized by macrophages. Perhaps the absence of necrotic masses indicates a constant activation of the immune system by an antigen that cannot be eliminated, which in turn may indicate autoimmune tissue damage [7].

The theory of autoimmune/inflammatory syndrome, induced by adjuvants (ASIA) was developed by Professor Y. Shoenfeld. This new data opened up new ways of understanding the pathogenesis of sarcoidosis [11,12]. 

## 2. Autoimmune/Inflammatory Syndrome Induced by Adjuvant Signs in Sarcoidosis

A certain similarity of ASIA and sarcoidosis as a genetically mediated disease with the development of immunological disorders under the influence of trigger factors that raises the question of their connection. This approach allows us to identify autoimmune features in the pathogenesis of this disease, which may bring us closer to understanding it.

### 2.1. Major Criteria

Major criteria include the exposure to external factors (infections, vaccines, silicone, or adjuvant). The clinical manifestations (from months to several years) develop before the provoking factor.

Some studies suggest that prolonged contact with inorganic dust, silicates, and print production is associated with the development of sarcoidosis [13,14,15]. Under laboratory conditions, carbonaceous materials, including multi-walled carbon nanotubes, have been shown to induce the development of granulomas in lungs with the formation of multi-nucleated giant cells in mouse models [16,17,18].

An important role is played by the implantation of various substances, such as silicone, which is widely used in the creation of breast implants, artificial joint shunts for patients with hydrocephalus, catheters, in rhinoplasty and as a part of the dermal fillers. A study by A. Watad that compared 24,651 patients with silicone implants to 98,604 patients without the presence foreign bodies, showed an increased risk of autoimmune diseases (OR 1.21, 95% CI 1.17–1.26) in female patients with silicone implants, having the strongest associations with sarcoidosis, Sjögren’s disease and systemic scleroderma [19,20]. A case of the development of an acute form of sarcoidosis, Löfgren’s syndrome were described after silicone implantation [21,22], as well as disease remission after the removal of implants. This may suggest a possible link between sarcoidosis and ASIA syndrome [20,23]. The role of silicone as a trigger in the development of sarcoidosis may be considered with both local (sarcoid granulomas in the skin at the site of silicone mammoplasty or subcutaneous injection) and, most importantly, systemic manifestations of sarcoidosis with the formation of granulomas in the lung tissue and hilar lymph nodes [24,25,26]. Cases of the development of pulmonary sarcoidosis with the involvement of the intracranial lymph nodes during the implantation of an intragastric silicone balloon are also described; the appearance of radiographic changes in the lung tissue was noted as early as 7 days after surgery [27].

When studying the infectious nature of sarcoidosis is actively discussed, as well asthe role of viruses, fungi and bacteria. Among which the most attention is paid to *Propionibacterium acnes* and *Mycobacterium tuberculosis* [8]. In numerous studies in patients with sarcoidosis the genetic material of this infection agents were found in sarcoid granulomas and in bronchoalveolar fluid. The activation of peripheral mononuclears in response to mycobacterial antigens has been shown, that indicated immune response against these microorganisms [28,29]. The effect of mycobacteria on the development of sarcoid granulomas has been shown in animal models (Lewis rats and C57BL/6 mice) [30]. In mouse models, the use of *M. tuberculosis* catalase peroxidase (mkatg) and *M. tuberculosis* A superoxide dismutase (MSODA) proteins [31,32], as well as various *P. acnes* proteins [33,34]. It showed the development of lung granulomatosis similar in histology to human sarcoid granulomas (the recruitment of macrophages and CD4 + T and CD45/B220+ B lymphocytes that stimulated the cytokine Th1 and chemokines (TNFα, IFNγ, MCP-1, IL12p40 and IL12p70)).

The association of the increase in the incidence of sarcoidosis with the presence of fungi in the dust was noted, and in vitro experiments showed the response of peripheral mononuclears when they were stimulated by antigens. These mold fungi were found in buildings where office employees worked [35,36].

One of the main trigger factors in ASIA is the adjuvants contained in vaccines. The post-vaccine development of juvenile sarcoidosis has been described in children after BCG administration, most commonly at the site of vaccine administration [37,38,39]. There have been published clinical cases with the development of similar to changes in sarcoidosis after the administration of varicella zoster virus vaccine (Shingrix) [40]:the tattooed skin [41]; [42]; the influenza vaccine; the rubella virus vaccine in a patient with primary immune deficiency [43].

Cases of sarcoid granuloma development in cancer patients deserve special attention. Therefore, cases of sarcoidosis after administration of a vaccine based on dendritic cells [44] and various peptides of melanoma cells [45] have been described.

A case of sarcoid mediastinal lymphadenopathy has also been described, presumably after vaccination against COVID-19 [46].

A distinction must be made between the terms “sarcoidosis” and "sarcoid reaction". The latter is characterized by the formation of epithelioid cell granulomas in organs and tissues, in some cases followed by similar clinical symptoms. The main difference between the sarcoid reaction and sarcoidosis is the presence of infectious diseases (i.e., tuberculosis, leishmaniasis, leprosy), tumors (i.e., prostate, breast, lung, colorectal cancer, ovarian and soft tissue tumors) or other trigger factors. The symptoms of sarcoid reaction are reversed upon the removal of the trigger factors. In accordance with this definition, it is possible to assume a link between the sarcoid reaction and ASIA syndrome. This does not exclude the possibility that in sarcoidosis there is also a trigger factor that cannot be clearly identified and removed [47,48].

Therefore, the supposed triggers of sarcoidosis and sarcoid reactions can be biological agents and substances of organic and inorganic origin, whose role in the pathogenesis of sarcoidosis, according to the data analysis, cannot be denied (Table 1).

At the same time, according to epidemiological studies, the influence of a single provoking factor has not been proven. It is assumed that different factors affect the immune system in different ways, stimulating the immune response and leading to the development of sarcoidosis, including the induction of autoimmunity, with genetic factors playing an important role [49,50,51].

#### 2.1.1. Appearance of at Least One of the following “Typical” Clinical Manifestations

Clinical manifestations of subacute and chronic sarcoidosis may be either absent or very diverse, and associated with lesions of various organs and nonspecific. Up to 50% of patients with pulmonary sarcoidosis has dry cough, dyspnea, and chest pain. Among the nonspecific symptoms, the most common are arthralgia, fatigue (up to 50–70% of patients), chest pain, muscle pain, night sweats, and weight loss. Chronic sarcoidosis is associated with the development of fibrosis (pulmonary and extrapulmonary), and pulmonary arterial hypertension. Adverse prognosis is also associated with the development of lupus pernio, chronic uveitis, hypercalcemia, nephrocalcinosis, cystic bone lesions, and myocardial damage [52,53].

The development of small fiber neuropathy (SFN) in sarcoidosis deserves special attention. Most often patients complain of burning paresthesias, numbness, and dysfunctions of the cardiovascular and musculoskeletal system and gastrointestinal tract, which is combined with a decrease in intraepidermal nerve fiber density in the biopsy material [54]. SFN is often found in various autoimmune diseases, which may indicate autoantibody-mediated nerve damage [55].

A more detailed description of the most common clinical manifestations associated with lesions of various organs and a comparison with the clinical manifestations of autoimmune diseases is presented in Table 2.

Sarcoidosis often occurs in conjunction with other autoimmune diseases. The main ones are:

- ankylosing spondylitis, granulomatous pulmonary disease, sacroiliitis and the HLA-B27 genotype typical for spondyloarthritis, which were detected in patients [56,57]
- lupus erythematosus [58];
- Sjogren’s syndrome [59];
- primary biliary cirrhosis, Crohn’s disease, antiphospholipid syndrome, and idiopathic pulmonary fibrosis [60,61]
- subclinical and clinical hypothyroidism, antithyroid autoantibodies [overall antithyroid peroxidase antibodies (TPOAb)], and, in general, thyroid autoimmunity [62];

Such cases of comorbidity show the possibility of a common pathogenesis of these diseases and confirm the idea of multi-autoimmune diseases [63].

#### 2.1.2. Typical Biopsy

The central part of a granuloma is composed of macrophages, modified macrophages, epithelioid cells, and giant cells, with CD4+ T-lymphocytes between them [64,65]. The peripheral part of a granuloma is predominantly occupied by CD8+ T lymphocytes, fibroblasts, and macrophages and fibrocytes. B lymphocytes are usually very rare in a granuloma [66]. Central fibrinoids may be found [7]. It is important to note the features of lymphocytic infiltration and the general state of the immune system in sarcoidosis which are similar to some autoimmune diseases [67]. In the presence of autoimmune processes, impaired memory and “naïve” B-cells distribution [68,69], an imbalance of T-helpers and T-follicular helpers towards an increase in the number of T-h17, T-fh17, Th2, a decrease in T-h1, T-regulatory cells [70], an imbalance between subpopulations of Tfh cells and regulatory Tfr [71,72], elevated levels of short-lived cells, and highly differentiated CD8 + T lymphocytes are observed [73].

Thus, in patients with neurosarcoidosis, a high CD8 + T-lymphocytes number was found during the immunohistochemical examination of granulomas in autopsies of the meninges and brainstem [74]. And an increase in the number of mature CD8 + CD56 + T cells, the relative content of which was almost three times higher than the value in the control group [75]. 

In addition to CD8 + T-lymphocytes, an increase in CCR4 + CD4 + cell numbers in the peripheral blood was found, as well as an increase in the concentration of the chemokine ligand for the chemokine receptor CCL17 in the blood serum of sarcoidosis patients and locally in the focus of granuloma formation [76]. Excessive Th2 activation in patients with sarcoidosis was also confirmed by the data on an increase of IL-13 mRNA expression, one of the key Th2 cytokines [77]. Moreover, in experiments on laboratory animals [78] and in the analysis of tissue samples obtained from patients [79], the overproduction of Th2 cytokines, and the activation and differentiation of tissue macrophages towards M2 was shown. M2 macrophages contribute to the development and maintenance of foci of chronic inflammation in tissues, the formation of granulomas, and the foci of fibrosis.

There is an assumption about the role of the disturbance of the Th1 and Th17 ratio in the formation of granulomas in sarcoidosis [80]. Most researchers have noted increased IL-6, IL-17, IL-22, IFN-γ and CCL20, synthesized by Th17 in the serum of patients [81], and increased levels of these cytokines, the number of cells involved in their production in the fluid of bronchoalveolar lavage (BAL), and granulomatous tissue [82].

T regulatory cells are immune suppressors and protect against the development of autoimmune diseases. In sarcoidosis, a paradoxical reaction is observed: in the focus of inflammation (in the BAL), a decrease in the number of cells was shown, while in the peripheral blood, to the contrary, an increase was shown. Huang et al. revealed an almost three-fold decrease in the relative number of T-regs in the blood of patients with initially diagnosed sarcoidosis compared with a group of conventionally healthy donors [80]. Furthermore, in many studies, a decrease in the level of the transcription factor T-reg FOXP3 in BAL was noted, which indicates a decrease in cell function [83]. Along with the anti-inflammatory cytokines TGF-β and IL-10, an important and relatively poorly studied soluble Treg product is adenosine, which has a pronounced suppressive effect on a wide range of cells of innate and adaptive immunity. [84]. When comparing the level of expression of CD39 on regulatory T-cells of peripheral blood, it turned out that the relative content of CD39 + cells is increased both in patients with acute onset and in primary chronic sarcoidosis [85]. It is likely that the formation of anti-inflammatory adenosine, which is involved in the suppression of hypersensitive immune responses, is one of the key and most common mechanisms of immunosuppression in both acute and chronic sarcoidosis.

In the study of humoral immunity, it was shown that in patients with sarcoidosis, the amount of B-cell-activating factor (BAFF) is significantly higher and, accordingly, Ig-producing B-cells in the lung tissues, while in the blood the level of B-cells in sarcoidosis, corresponded to the number in healthy people or was below normal [86]. At the same time, a disbalance of B-cell subtypes was observed. A decrease in the number of memory B-cells, an increase in the number of B-regulatory cells producing IL-10, and hypergammaglobulinemia, correlating with an increase in immunoglobulins in the peripheral blood, were noted in BAL [87]. Hypergammaglobulinemia has been shown in several studies, but no association with disease severity has been found [88].

According to a comprehensive analysis of B- and T-lymphocytes [89,90], sarcoidosis patients showed an increase in the number of naive B cells, a decrease in memory B cells, and an increase in the number of CD24 +++ CD38 +++ and CD5 + CD27– B cells with regulatory functions, which have been described for many autoimmune diseases. A significantly higher proportion of CXCR5-expressing CD45RA—CCR7 + Th cells was found in patients with sarcoidosis compared with healthy controls, representing an expansion of this subpopulation of memory Th cells in disease.

A comparison of T- and B-lymphocyte subpopulations in sarcoidosis and autoimmune diseases is presented in Table 3.

We noted that the lymphocyte count can serve as a prognostic factor. The higher number of CD4 cells and a higher CD4/CD8 ratio was observed in radiologically improved patients and responded better to treatment. The increasing number of Th17 cells can be predictive for progressive sarcoidosis and may help in the selection of patients with an increased risk of lung fibrosis [91]. Low neutrophil count in the BAL fluid was associated with higher chances of spontaneous recovery [92].

### 2.2. Minor Criteria

#### 2.2.1. The Appearance of Autoantibodies or Detection of Antibodies against the Suspected Adjuvant

At the moment, no specific autoantibodies have been identified in sarcoidosis; however, in various studies, autoantibodies to a wide range of antigens have been found in patients, which is very characteristic of ASIA syndrome (Table 4). The Shi TY study showed that patients with sarcoidosis were more likely to have autoantibodies despite no presence of autoimmune disease [93].

According to a comprehensive analysis of antibodies and subpopulations of B-lymphocytes [89], patients with sarcoidosis showed an increase in anti-MCV (anti—mutated citrullinated vimentin) of more than 14 U/mL, an increase in “naive” B-cells, and a decrease in memory B-cells, and an increase in CD24 +++ CD38 ++ + B- cells. Based on these data, the research team developed a formula for calculating the *DS* index (1), the use of which suggests the presence of autoimmune inflammation. With an index value of more than seven units (AUC = 0.889) and a sensitivity of 80.56% and a specificity of 93.33%, it is possible to assume the presence of sarcoidosis.

DS = (B-naïve/B-memory*(CD38-B-cells + CD5-B-cells)*[anti-MCV]/100 (1)

(B-naive\B-memory)—the index of the humoral immune response activity;

(CD38-B-cells + CD5-B-cells)—the total number of regulatory B cells (IL-10 synthesis);

[anti-MCV]—the concentration of anti-MCV, characterizing the presence of autoimmune reactions.

The use of the formula can make it possible to identify patients with the presence of autoimmune inflammation in the pathogenesis of the development of sarcoidosis, which can serve as a recommendation to treat them with immunosuppressive therapy and can serve as a prognostic or predictive marker in assessing the course of the disease and therapy efficacy.

Autoantibodies to vimentin and its modifications have been found in various studies in patients with sarcoidosis [89,99,100,101]. There is a hypothesis among researchers about the molecular mimicry of vimentin and mycobacterial proteins [102,103,104]. The heat shock proteins Mtb-HsP60, Mtb-HsP65, and catalase (mKatG) are considered to participate in cross-reactions with human peptides [29,105]. However, no publications on the comparison of the structure of vimentin and mycobacterial proteins have been published, which provides new prospects for future research. Serum amyloid A (SSA) is considered to promote TLR2 activation and sustained granulomatous inflammation [32].

#### 2.2.2. Specific HLA (i.e., HLA DRB1, HLA DQB1)

According to recent studies, HLA genotypes are one of the main genetic predisposing factors for autoimmune disease development. Associations with the HLA-I class, HLA-II, class and non-HLA genes have been identified, e.g., BTNL2, C4, C6 orf10, HSPA1L, LTA, NOTCH4, TAP2, TNF, and VEGF [106].

Our greatest interest was in the HLA-DRB1 genotypes, which are associated with the development of many autoimmune diseases. According to the analysis of the distribution of HLA-DRB1, -DQ1 genotypes in different populations [107] the main predisposing genotypes in the development of sarcoidosis can be identified:

- Löfgren syndrome; HLA-DRB1*01/03, HLA-DQB1*02.01
- The chronic form of HLA-DQB1*06.02, HLA-DRB1*07/14/15
- The protective effect of HLA-DRB1*01/04

More detailed data on the distribution of genotypes are presented in Table 5.

#### 2.2.3. Immunosuppressive Therapy

It should be noted that most cases of newly diagnosed sarcoidosis do not require treatment, and only dynamic observation is recommended because in half of the patients the disease undergoes spontaneous remission within several years [123]. This association is most clearly manifested in sarcoid reactions, one of the features of which is the regression of changes after the removal of the provoking factor.

However, in some patients, the disease may be accompanied by a progressive course with the formation of insufficiency of the affected organ, which requires therapy. At the same time, despite the treatment, in a significant number of patients the sarcoidosis will turn into a chronic and progressive form, and 6–7% will be fatal [123]. One of the reasons for the complexity of the therapeutic approach is the lack of unified prognostic markers of the course of the disease, as well as the lack of clarity of the etiological factors and the incomplete understanding of the pathogenesis of the disease, which complicates the development of specific treatment methods.

One of the features of sarcoidosis that links it to autoimmune diseases is the response to the therapy commonly used to treat these diseases [124,125]. No drug for the treatment of sarcoidosis has been approved by the FDA because of the unknown etiology of the disease. This determines the need for additional research on autoimmune pathology in sarcoidosis.

At the moment, the first line of therapy includes glucocorticosteroids (prednisolone and methylprednisolone) [126]. Alternatively, immunosuppressive, cytotoxic and antimalarial agents can be used. The most commonly prescribed second-line drug is methotrexate, and other cytostatics such as leflunomide, azathioprine, and combinations of methotrexate and leflunomide are also possible. Azathioprine affects the synthesis of RNA and DNA thereby suppressing the proliferation of lymphocytes, while exerting a greater effect on cellular immunity than humoral immunity. The exact mechanism by which azathioprine may affect sarcoidosis is unclear [127].

In case of the ineffectiveness of the second line of therapy, the next step is to prescribe biological therapy drugs [128]. Targeted TNF therapy was the first biological therapy prescribed to patients with sarcoidosis because TNF-α is secreted by macrophages in patients with active sarcoidosis, and plays a key role in the development of sarcoid granuloma. TNF-targeted therapy includes monoclonal antibodies directed against TNF (infliximab, adalimumab, golimumab), a recombinant protein that connects the TNF receptor to the constant end of an IgG1 antibody (etanercept) and a pegylated Fab fragment of a humanized anti-TNF-α monoclonal antibody (certolizumab) [129,130]. The drugs have different efficacy in different forms of sarcoidosis; however, unfortunately, the data on their use are still insufficient to develop a clear algorithm [131,132,133].

In the case of contraindications or ineffectiveness, it is possible to use off-label hydroxychloroquine, as well as mycophenolate, cyclophosphamide, or rituximab (these prescriptions are based on experience in the treatment of other immunological lung diseases, with sarcoidosis not fully understood [134,135]).

## 3. Sarcoidosis Models

Despite the long-term studies on the pathogenesis of sarcoidosis, there are still no reliable models of the disease [136], which would allow for the demonstration of the influence of the trigger factor, as well as the determination of the etiotropic therapy. In particular, when exposed to various antigens (peptides *M. Tuberculosis*, *P. Acne*, carbon nanoparticles) in mouse models, only pulmonary granulomas without fibrosis were obtained. Genetically modified mice developed multiple sites of granulomatosis (lungs, skin, liver, stomach, ganglia), which may indicate the peculiarities of the immune response in healthy mice, allowing for the more efficient elimination of pathogens [137]. It was also shown that the granulomas obtained in the experiments cannot exist without the continued influence of the adjuvant. The reviewers suggest that sarcoidosis patients are chronically exposed to one or more triggers that promote the development and maintenance of granulomas.

In a recent publications, it was shown in a mice model that in vimentin-immunization mice granulomas were found in the lung following intervenous challenge with vimentin-coated beads [138]. Sarcoidosis-like granulomas showed the presence of Langhans and foreign body multinucleated giant cells, CD4 T cells, and a heregeneus collection of MHC II positive and arnase 1 expressing macrophages.

The unambiguous conclusion of all existing reviews and studies devoted to the creation of a model of sarcoidosis is that in the present conditions it is impossible to achieve this goal, which determines the empirical approach to treatment.

## 4. Discussion

**Figure 1:**
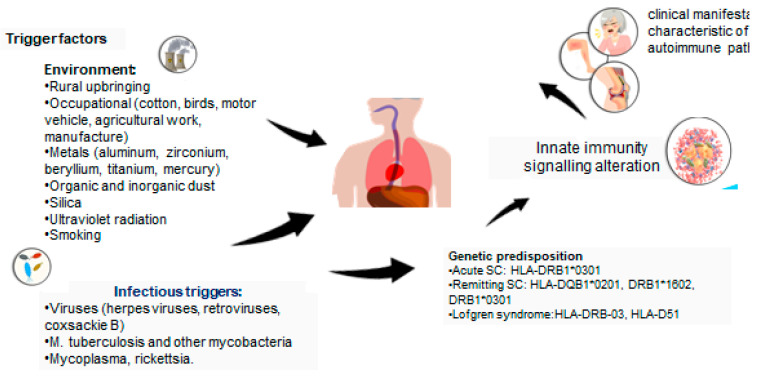
ASIA criteria in sarcoidosis.

According to the analysis, it can be assumed that there are several autoimmune features in the concept of ASIA syndrome in sarcoidosis. The characteristic features confirming this fact include (Figure 1):

- connection with the HLA genotype;
- the impact of various trigger factors;
- improvement of the condition with the elimination of the trigger factor;
- immunological disorders in the form of cytotoxicity of lymphocytes upon stimulation with autoantigens, the presence of autoantibodies, an imbalance of subpopulations of T- and B-lymphocytes typical for many autoimmune diseases;
- clinical manifestations characteristic of autoimmune pathology;
- a histological picture of the disease;
- an association with other autoimmune diseases;
- the efficacy of immunosuppressive therapy.

However, despite certain associations between sarcoidosis and ASIA, it is impossible to unambiguously consider all variants of the course of sarcoidosis within the framework of this syndrome. Perhaps we should talk about different diseases or different “stages” of the course of sarcoidosis, depending on the continuing influence of the trigger factor. The majority of patients with sarcoidosis are prone to self-healing that can be associated not only with genetic and immunological characteristics.

**Figure 2:**
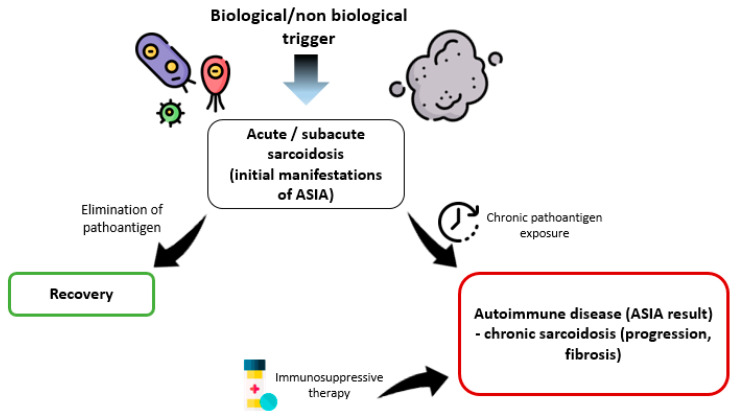
Stages of the course of sarcoidosis within ASIA syndrome.

With regard to the relationship between ASIA and sarcoidosis, it is important to identify a cohort of patients in whom self-healing does not occur, and the course of the disease becomes progressive with the development of fibrosis, organ failure, and the generalization of the process. We can assume that in this situation the pathoantigen is not eliminated, and a full-fledged autoimmune disease develops, requiring the appointment of immunosuppressive therapy, which fits into the concept and criteria of ASIA (Figure 2).

## 5. Conclusions

The analysis of the connection between ASIA and sarcoidosis suggests the need to continue research with studying the prognostic and predictive factors that determine the course of the disease, in addition to treatment strategies. Questions about the etiological factors, the creation of a model of sarcoidosis, and the justified prescription of immunosuppressives, including biological therapy, remain unanswered. And the relationship of sarcoidosis and ASIA, on the contrary, raises new ones.

## Figures and Tables

**Table 1:** Suspected organic and inorganic triggers of sarcoidosis development [13,14,15,16,17,18,19,20,21,22,23,24,25,26,27,28,29,30,31,32,33,34,35,36,37,38,39,40,41,42,43,44,45,46,47,48,49,50,51].

Biological Triggers	Non Biological Triggers
**Bacteria** (*M. tuberculosis*, *Nontuberculous Mycobacterium*, *P. acnes*) **Fungi** **Viruses** (human herpes viruses HHV6 and HHV8, retrovirus, cytomegalovirus, Coxsackie virus, rubella virus, adenovirus, SARS-CoV-2, etc.) **Vaccines** (tuberculosis, flu, cancer)	**Organic compounds** (pine pollen, clay, silicon, etc.) **Metal dust** (aluminum, zirconium, barium, cobalt, copper, titanium, gold, etc.) **Other inorganic compounds** (talc, photocopy toner powder, fiberglass, etc.)

**Table 2:** Clinical manifestations of sarcoidosis typical for autoimmune diseases [52,53,54,55].

Affected Organ	Percentage of Occurrence	Clinical Manifestations	Clinical Manifestations Typical for Autoimmune Diseases
Lymph nodes	95–98%	Lymphadenopathy	
General symptoms	up to 35%	Fever, unexplained weight loss, fatigue, sleep disturbances	Chronic fatigue, unrelieved sleep, sleep disturbances Fever, dry mucous membranes
Eyes	20–50%	Uveitis, retinal vascular changes, lacrimal gland enlargement	Uveitis
Musculoskeletal system	25–39%	Myositis, muscle weakness, arthralgia, and/or arthritis	Myalgia, myositis, muscle weakness Arthralgia and/or arthritis
Skin	25%	Papules, nodules, erythema nodosum, lupus pernio, granulomas in the area of tattoos, scars	Rash
Nervous system	10%	Cognitive dysfunction, small fiber neuropathy (burning paresthesias, numbness, dysfunctions of the cardiovascular, musculoskeletal system and gastrointestinal tract)	Neurological manifestations (especially associated with demyelination), cognitive disorders, memory impairment, small fiber neuropathy

**Table 3:** Features of lymphocytic populations in sarcoidosis and autoimmune diseases [64,65,66,67,68,69,70,71,72,73,74,75,76,77,78,79,80,81,82,83,84,85,86,87,88,89,90,91,92].

	Sarcoidosis		Autoimmune Diseases
	Number in Peripheral Blood	Number in BAL	
CD8 T cells	↑	↑	↑
CD4 T cells	↑	↑	↑
T reg suppression of immune responses	↑	↓	↓
Th17 cell proliferation activation	↑	↑	↑
Th 1 activation of cytotoxic immunity	n/a *	n/a *	↓
Th 2 B cell activation	↑	↑	↑
Memory B cells	↓	↓	↓
Naïve B cells	↑	↑	↑
Activated B cells	↑	↑	↑

* n/a—not available.

**Table 4:** The detection of various autoantibodies in sarcoidosis patients in various studies.

First Author’s Name, Year	Detected Autoantibodies	Diagnosis
Maddrey WC, 1970 [94]	antimitochondrial antibodies	sarcoidosis and chronic liver damage
Fagan EA, 1983 [95]		sarcoidosis and liver damage
Stanca CM, 2005 [60]		sarcoidosis and primary biliary cirrhosis
Weinberg I, 2000 [96]	antinuclear antibodies	sarcoidosis
	antibodies to deoxyribonucleic acid (DNA)	
Kobak S, 2014 [97,98]	antibodies to citrullinated cyclic peptide	
	rheumatoid factor	
Kinloch AJ, 2018 [99]	antibodies to vimentin	
A. Malkova, 2021 [89]	anti-MCV	

**Table 5:** Distribution of HLA-DRB1 genotypes in sarcoidosis patients.

Name of the First Author and Year	Patients with Sarcoidosis and Löfgren’s Syndrome;	The Statistically Approved HLA Genotypes		Country
		of Predisposition	Protective	
Berlin M, 1997 [108]	Sarcoidosis (*n* = 122)	DRB1*03, 17	-	Scandinavia
	Lofgren’s syndrome (*n* = 34)	DRB1*17	-	
	Chronic form (*n* = 57)	DRB1*14, 15	-	
Bogunia-Kubik K, 2001 [109]	Sarcoidosis (*n* = 53)	DRB1*03	DRB1*11	Poland
Foley, 2001 [110]	Sarcoidosis (*n* = 345)	DRB1*03	DRB1*01, 04	UK, Poland, Czech republic
Planck A, 2002 [111]	Lofgren’s syndrome (*n* = 19)	DR B1*17	-	Scandinavia
Rossman MD, 2003 [112]	Sarcoidosis (*n*= 948)	DRB1*1101, 1501, 1201	DRB1*0401, 0404, 0407, 1503	USA
Sharma SK, 2003 [113]	Sarcoidosis (*n* = 56)	DRB1*11, 14 (chronic form)	DRB1*07	India
Grunewald, 2010 [114]	Sarcoidosis (*n* = 724)	DRB1*03, 14, 15	DRB1*01, 03, 07	Scandinavia
Voorter CE, 2005 [115]	Sarcoidosis (*n* = 149)	DRB1*15:0101—severe form	-	The Netherlands
Papadopoulos KI, 2006 [116]	Sarcoidosis (*n* = 66)	DRB1*02, 14	-	Germany
Darlington P, 2011 [117]	Patients with sarcoidosis (*n* = 1000) who had symptoms associated with HS (Heerfordt’s syndrome) (*n* = 83)	DRB1*04 - uveitis	DRB1*04—system sarcoidosis	Sweden
da Costa CH, 2013 [118]	Sarcoidosis (*n* = 63)	DRB1*14	DRB1*15, DRB1*16	Brazil
Ozyilmaz E, 2014 [119]	Pulmonary sarcoidosis (*n* = 86), Extrapulmonary sarcoidosis (*n* = 46)	DRB1*15	DRB1*11—from extra-pulmonary	
Levin AM, 2015 [120]	Sarcoidosis (*n* = 1277)	DRB1*12, 11	DRB1*03	USA
Mortaz E, 2015 [121]	Pulmonary sarcoidosis (*n* = 51), Extrapulmonary sarcoidosis (*n* = 39)	DRB1*7—pulmonary DRB1 *12—Extrapulmonary	-	Iran
Yanardag H, 2017 [122]	Sarcoidosis (*n* = 74)	DRB1*07, 14, 15	-	Turkey

## Data Availability

All data generated or analyzed in this study are included in this published article).
